# Prognostic Implications of Right Atrial Dysfunction in Adults With Pulmonary Atresia and Intact Ventricular Septum

**DOI:** 10.1016/j.cjcpc.2021.11.001

**Published:** 2022-02-02

**Authors:** Alexander C. Egbe, William R. Miranda, Heidi M. Connolly

**Affiliations:** Department of Cardiovascular Medicine, Mayo Clinic Rochester, Rochester, Minnesota, USA

## Abstract

**Background:**

Pulmonary atresia with intact ventricular septum is associated with significant morbidity and mortality, but there are limited data to guide risk stratification in this population. The purpose of this study was to assess the role right atrial (RA) strain indices for prognostication in this population.

**Methods:**

This is a retrospective study of adults (aged ≥18 years) with pulmonary atresia with intact ventricular septum and biventricular repair who underwent echocardiogram (2003-2019). RA reservoir strain was used as the primary metric of RA function, and RA dysfunction was defined as RA reservoir strain <31%. Clinical outcomes were assessed using 4 different indices: (1) functional impairment (New York Heart Association II-IV); (2) hepatorenal dysfunction (model for end-stage liver disease excluding international normalized ratio score >11); (3) incident atrial arrhythmias/heart failure hospitalization; (4) heart transplant/cardiovascular death.

**Results:**

Of the 43 patients in the study, RA strain imaging was feasible in 95%, and RA dysfunction was present in 95%. Of the 43 patients, 67% and 49% had functional impairment and hepatorenal dysfunction, respectively; 44% developed incident atrial arrhythmia/heart failure hospitalization and 14% died during follow-up. RA reservoir strain was independently associated with all indices of clinical outcomes.

**Conclusion:**

Collectively, these data suggest that RA strain imaging was feasible in almost all patients and can be used for risk stratification in this population. There was a high prevalence of comorbidities including hepatorenal dysfunction. Further studies are needed to determine the prognostic implications of hepatorenal dysfunction (a previously unrecognized complication), and whether using RA function indices for clinical decision making will lead to improved outcomes in this population.

## Résumé

**Contexte:**

L’atrésie pulmonaire à septum ventriculaire intact est associée à une morbidité et à une mortalité importantes, mais il existe peu de données pour guider la stratification des risques au sein de la population touchée. L’étude dont il est question dans notre article visait à évaluer le rôle des indices de déformation (strain) atriale droite (AD) dans la formulation d’un pronostic en présence de cette cardiopathie.

**Méthodologie:**

Nous présentons ici une étude rétrospective portant sur un groupe d’adultes (âgés de 18 ans ou plus) atteints d’atrésie pulmonaire à septum ventriculaire intact qui avaient subi une réparation biventriculaire et une échocardiographie (2003-2019). La déformation AD pendant la phase réservoir a été retenue comme mesure principale de la fonction AD, et la dysfonction AD correspondait à une déformation AD < 31 % pendant la phase réservoir. Les résultats cliniques ont été évalués à l’aide de quatre indices distincts : (1) déficience fonctionnelle (classes II à IV de la New York Heart Association); (2) dysfonction hépatorénale (modèle d’hépatopathie en phase terminale excluant un rapport international normalisé > 11); (3) épisodes d’arythmie auriculaire incidente/hospitalisation pour cause d’insuffisance cardiaque; (4) transplantation cardiaque/décès d’origine cardiovasculaire.

**Résultats:**

Au sein du groupe de 43 patients à l’étude, l’imagerie de la déformation AD était réalisable et une dysfonction AD existait dans 95 % des cas respectivement. Parmi les 43 patients, 67 % présentaient une déficience fonctionnelle et 49 %, une dysfonction hépatorénale; 44 % présentaient une arythmie auriculaire incidente ou avaient été hospitalisés pour insuffisance cardiaque et 14 % étaient décédés au cours de la période de suivi. La déformation AD pendant la phase réservoir était indépendamment associée à tous les indices des résultats cliniques.

**Conclusion:**

Globalement, ces données donnent à penser que l’imagerie de la déformation AD était réalisable chez presque tous les patients et qu’elle peut servir à la stratification des risques au sein de la population considérée. La prévalence de maladies concomitantes était élevée, notamment en ce qui concerne la dysfonction hépatorénale. D’autres études devront être menées pour cerner l’incidence pronostique de la dysfonction hépatorénale (complication non reconnue antérieurement) et pour déterminer si l’utilisation d’indices de la fonction AD lors de la prise de décisions cliniques se traduira par de meilleurs résultats chez les patients.

Pulmonary atresia with intact ventricular septum (PA-IVS) is characterized by the absence of a direct communication between the right ventricle (RV) and the pulmonary artery, and it is associated with varying degree of RV and tricuspid valve hypoplasia.^1^^,^^2^ The patients with RV-dependent circulation typically undergo univentricular repair, whereas those without RV-dependent circulation undergo biventricular or 1.5 ventricular repair depending of the severity of RV hypoplasia.^3^, ^4^, ^5^ Long-term outcomes studies show that adults with PA-IVS who underwent biventricular repair in childhood have significantly impaired exercise capacity, high burden of atrial arrhythmias, and cardiovascular death, and the long-term outcomes in these patients are similar to those in patients who received univentricular or 1.5 ventricular palliation.^3^, ^4^, ^5^, ^6^, ^7^ This is attributed to severe RV diastolic dysfunction, which is often exacerbated by concomitant tricuspid and pulmonary valve disease.^8^^,^^9^ There are limited data to guide risk stratification in this population because, in contrast to other patients with RV outflow tract disease, the underlying haemodynamic problem is impaired RV compliance rather than RV volume overload.^10^^,^^11^

Speckle-tracking imaging of the right atrium (RA) provides a comprehensive assessment of right heart diastolic function, because it reflects the interaction between RA relaxation and contractility, and RV relaxation and compliance.^12^^,^^13^ Changes in RA function correlate with changes in invasive haemodynamic indices of right heart function, thus making RA strain imaging an ideal clinical tool for risk stratification.^12^^,^^13^ Because PA-IVS is characterized by RV diastolic dysfunction, which typically deteriorates over time,^8^^,^^9^ RA strain imaging can potentially be used for prognostication in this population, but such data are currently lacking. The purpose of this study was to assess RA function using speckle-tracking imaging in adults with PA-IVS and biventricular repair, and to evaluate the relationship between RA function indices and clinical outcomes. We hypothesized that RA function, as measured by RA reservoir strain, will be independently associated with clinical outcomes.

## Methods

### Study population

This is a retrospective study of adult patients (aged ≥18 years) with PA-IVS and biventricular repair who received care at Mayo Clinic from January 1, 2003, to December 31, 2019. Patients who underwent univentricular or 1.5 ventricular repair were excluded. The Mayo Clinic Institutional Review Board approved the study protocol. The electronic medical records were reviewed to retrieve surgical, clinical, laboratory, and invasive haemodynamic data.

### Echocardiography

**Figure 1:**
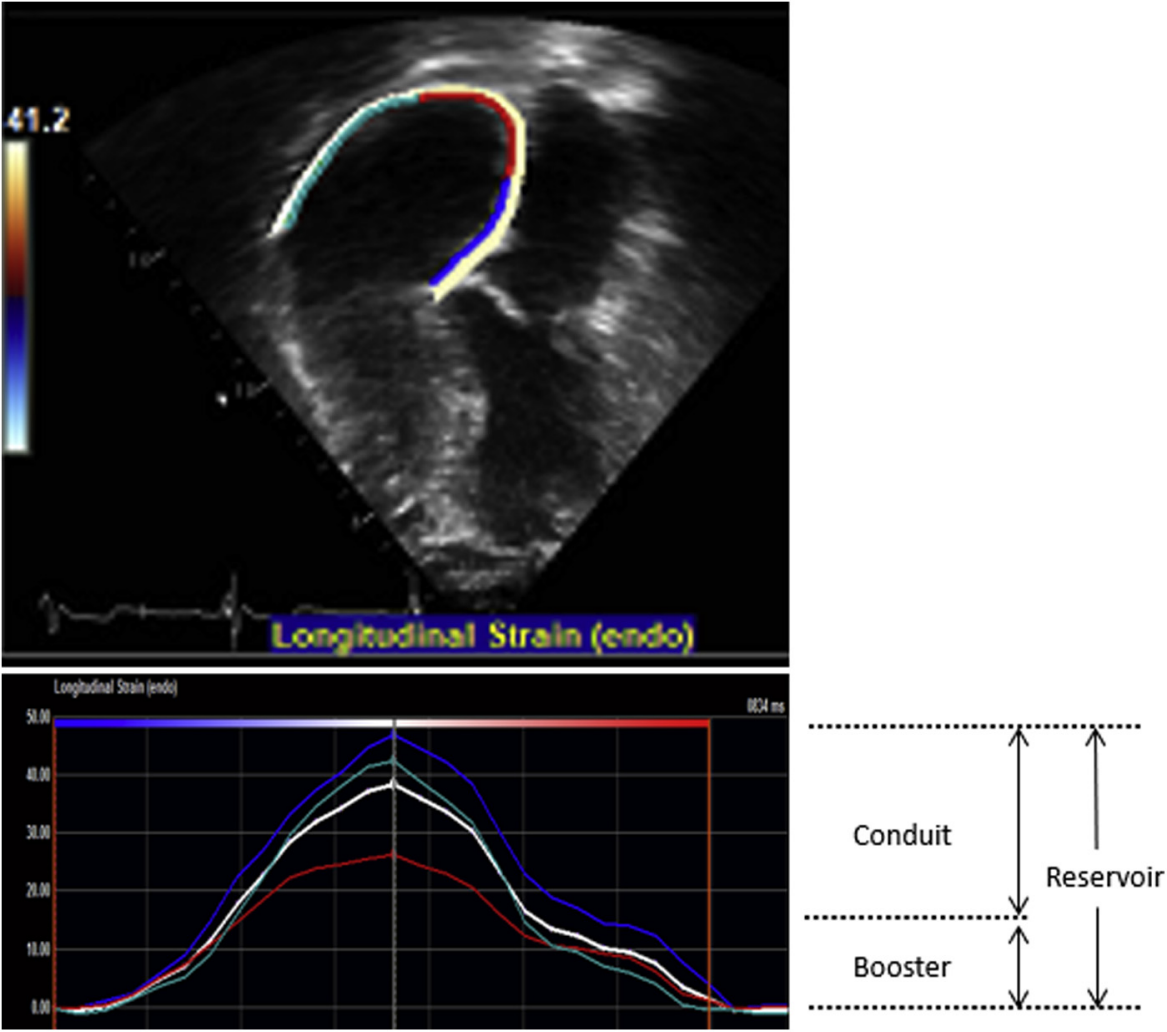
2D and graphical tracing of right atrial strain, which is an average of strain indices from the left wall, right wall, and roof of the right atrium. Reservoir strain is measured at the end of ventricular systole, booster strain is measured at the end of atrial systole, and conduit strain is calculated as the difference between reservoir strain and booster strain.

The first echocardiogram performed within the study period was reviewed, and offline analyses and measurements were performed by a single research sonographer. The procedural techniques for 2D, Doppler, and speckle-tracking imaging for the assessment of right heart remodelling have been described in previous studies.^12^, ^13^, ^14^ In brief, 3-beat cine-loop clips of the RA and RV obtained from RV-focused apical 4-chamber views were exported (DICOM) and then analyzed offline using TomTec (TomTec Imaging Systems, Unterschleissheim, Germany). The RA endocardial border was manually traced at ventricular end-systole in the apical 4-chamber view, starting at the lateral tricuspid valve annulus, along the endocardial border of the RA lateral wall, RA roof, RA septal wall, and ending at the septal tricuspid valve annulus (Fig. 1). Adequate tracking by the software was visually verified and retraced if necessary, until adequate tracking was achieved. We excluded the cine-loop clips with poor visualization or poor tracking of >1 atrial segment or significant foreshortening of the RA or RV. The software divided the RA into 3 separate segments (RA lateral wall, RA roof, and RA septal wall) and generated the RA strain curves. RA reservoir, conduit, and booster strain were derived from these curves. For the purpose of this study, we chose RA reservoir strain as the primary metric of RA function based on previous studies showing its superior prognostic performance as compared with RA conduit and booster strain.^12^^,^^13^ We defined RA dysfunction as RA reservoir strain <31%.^14^

Other indices of right heart function were also assessed, and these indices include RA volume (RA enlargement was defined as RA volume index >27 mL/m^2^ in females or >32 mL/m^2^ in males); RA pressure (RA hypertension was defined as echo-derived estimated RA pressure ≥8 mm Hg); RV global longitudinal strain (RV systolic dysfunction defined as RV global longitudinal strain >−18%); RV end-diastolic area (RV enlargement defined as RV end-diastolic area >25 cm^2^); and RV free wall thickness (RV hypertrophy defined as wall thickness >5 mm).^14^^,^^15^

### Clinical outcomes

Clinical outcomes were assessed using 4 different indices: (1) functional status was assessed using the New York Heart Association (NYHA) classification; (2) hepatorenal dysfunction was assessed using the model for end-stage liver disease excluding international normalized ratio (MELD-XI score), and significant hepatorenal dysfunction was defined as the MELD-XI score >11 based on published data;^16^ (3) incident atrial arrhythmias (atrial flutter/tachycardia and atrial fibrillation) and heart failure hospitalization during follow-up; and (4) heart transplant or cardiovascular death. The assessment of functional status and hepatorenal function was based on clinical assessment and comprehensive metabolic panel performed within 3 months from the time of the baseline echocardiogram (ie, first echocardiogram performed within the study period). The occurrence of incident atrial arrhythmias was ascertained by review of electrocardiograms and Holter monitors, and heart transplant/cardiovascular death was ascertained by review of medical records from the time of the baseline echocardiogram to the last clinical encounter or end of study period.

### Exploratory analysis

Exploratory analysis was performed to determine whether RA function (RA reservoir strain) declined over time and whether the temporal change in RA function was associated with composite endpoint of incident atrial arrhythmia/heart failure hospitalization. For this analysis, we measured RA reservoir from echocardiograms performed 36 months (24-48 months) after the baseline echocardiogram.

### Statistical analysis

The correlations between RA reservoir strain and clinical outcomes were assessed using linear, logistic, and Cox regression as appropriate. Follow-up duration was calculated from the date of baseline echo until the end of the study period (December 31, 2019) or until study censure as appropriate. We adjusted for right heart indices (RA volume, RA pressure, RV systolic pressure, and RV global longitudinal strain), left heart indices (left ventricular longitudinal strain and cardiac output), valve function (tricuspid and pulmonary regurgitation), and demographic indices (sex, current age, and age at the time of repair) using stepwise forward selection with a *P* value of <0.1 required for a variable to remain in the model. We corrected for missing data using the single conditional imputation method. All statistical analyses were performed with JMP software (version 14.1.0; SAS Institute Inc, Cary, NC), and a *P* value of <0.05 was statistically significant.

## Results

### Baseline characteristics

A total of 43 patients met the study inclusion criteria. The mean age at the time of the baseline echocardiogram was 26 ± 5 years, and the mean age at the time of complete repair (implantation of a pulmonary valve conduit) was 4 (1-9) years (Table 1). All patients have pulmonary valve conduit implantation (inclusion criteria). All patients had palliative systemic-to-pulmonary shunt (Blalock-Taussig shunt n = 41 and Waterston shunt n = 2), and the median age at the time of palliative shunt was 9 (2-23) days. One of the patients underwent stent implantation to maintain the patency of the ductus arteriosus at 2 days of age but subsequently underwent surgical implantation of a Blalock-Taussig shunt at 8 days of age because of persistent cyanosis. None of the patients underwent transcatheter perforation or stenting of the RV outflow tract.

### Haemodynamic indices

The assessment of RA reservoir strain was feasible in 41 (95%) patients. Of these 41 patients, RA enlargement and RA hypertension were present in 41 (100%) and 40 (98%) patients, respectively (Table 2). There was a correlation between RA reservoir strain and RA volume (*r* = −0.36, *P* = 0.006), estimated RA pressure (*r* = −0.43, *P* < 0.001), estimated RV systolic pressure (*r* = −0.32, *P* = 0.03), and RV global longitudinal strain (*r* = 0.38, *P* = 0.01). Of note, RV enlargement and RV systolic dysfunction were present in 12 (28%) and 18 (42%) patients, respectively, whereas RV hypertrophy was present in 100% of the patients.

Of the 43 patients, 16 (37%) had cardiac catheterization data, and the median interval between echocardiogram and cardiac catheterization was 6 (3-35) days. There was a correlation between RA reservoir strain and invasively measured RA pressure (*r* = −0.51, *P* < 0.001) and invasively measured RV systolic pressure (*r* = −0.38, *P* = 0.02), but not pulmonary vascular resistance (*r* = −0.12, *P* = 0.4).

### Clinical outcomes

Of the 43 patients, 14 (33%), 11 (26%), 13 (30%), and 5 (12%) were in NYHA functional class I, II, III, and IV, respectively. RA reservoir strain was associated with functional impairment (NYHA II-IV) (odds ratio: 0.91 [0.87-0.95], *P* = 0.006), after adjustment for other right heart indices (RA volume, RA pressure, RV systolic pressure, and RV global longitudinal strain), left heart indices (left ventricular longitudinal strain and cardiac output), valve function (tricuspid and pulmonary regurgitation), and demographic indices (sex, current age, and age at time of repair) (Table 3). The assessment of the MELD-XI score was feasible in all patients. The median MELD-XI score was 10.9 (10.3-14.7), and 21 (49%) patients had significant hepatorenal dysfunction (MELD-XI > 11). RA reservoir strain was independently associated with the MELD-XI score (β ± standard error: −0.31 ± 0.18, *P* < 0.001) (Table 4).

**Table 1:** Baseline characteristics (n = 43)

Age (y)	26 ± 5
Male, n (%)	18 (42)
Body mass index (kg/m^2^)	26 ± 5
Prior palliative shunt, n (%)	43 (100)
Age at time of complete repair (y)	4 (1-9)
Number of cardiac surgeries	2 (2-4)
Number of conduit replacements, n (%)	
1	22 (51)
2	16 (37)
3	5 (12)
RV-coronary artery fistula, n (%)	9 (21)
RV-dependent coronary circulation	0
Comorbidities, n (%)	
Hypertension	1 (2)
History of atrial fibrillation	8 (19)
History of atrial flutter	5 (12)
Laboratory Data	
NT proBNP (pg/mL)	197 (109-641)
Haemoglobin (g/dL)	13.2 ± 1.7
Estimated GFR (mL/min/1.73 m^2^)	81 ± 19
Medications, n (%)	
Loop diuretics	11 (26)
Beta-blockers	7 (16)
ACEI/ARB	5 (12)
Aldosterone antagonist	6 (14)

ACEI, angiotensin converting enzyme inhibitor; ARB, angiotensin II receptor blockers; GFR, glomerular filtration rate; NT proBNP, N-terminal pro-brain natriuretic peptide; RV, right ventricle.

**Table 2:** Haemodynamic indices

Echocardiography	
Right heart	
RA volume index (mL/m^2^)	39 ± 14
RA reservoir strain (%)	23 ± 9
RA booster strain (%)	13 ± 6
RA conduit strain (%)	10 ± 4
Estimated RA pressure (mm Hg)	13 ± 5
RV global longitudinal strain (%)	−19 ± 4
RV wall thickness (mm)	8 ± 2
RV end-diastolic area (cm^2^)	21 ± 7
RV end-systolic area (cm^2^)	13 ± 4
RV fractional area change (%)	39 ± 8
RV s′ (cm/s)	9 ± 3
RV e′ (cm/s)	5 ± 2
≥Moderate tricuspid regurgitation, n (%)	7 (16)
Tricuspid regurgitation velocity (m/s)	3.1 ± 0.6
Estimated RV systolic pressure (mm Hg)	47 ± 9
≥Moderate pulmonary regurgitation, n (%)	18 (41)
Pulmonary valve mean gradient (mm Hg)	10 ± 6
Left heart	
LV end-diastolic volume index (mL/m^2^)	49 ± 9
LV end-systolic volume index (mL/m^2^)	22 ± 5
LV stroke volume index (mL/m^2^)	36 ± 7
LV ejection fraction (%)	58 ± 9
LV global longitudinal strain (%)	21 ± 6
Lateral mitral E/e′	6 ± 3
Cardiac catheterization (n = 16)	
RA pressure (mm Hg)	16 ± 7
RV systolic pressure (mm Hg)	43 ± 12
RV end-diastolic pressure (mm Hg)	15 ± 8
PA systolic pressure (mm Hg)	31 ± 13
PA diastolic pressure (mm Hg)	15 ± 10
PA mean pressure (mm Hg)	21 ± 11
PA wedge pressure (mm Hg)	13 ± 4
Qp index (L/min/m^2^)	2.58 ± 0.91
Qs index (L/min/m^2^)	2.51 ± 0.86
Pulmonary vascular resistance index (WU m^2^)	3.22 ± 1.03
Aortic systolic pressure (mm Hg)	112 ± 17

LV, left ventricle; PA, pulmonary artery; Qp, pulmonary blood flow; Qs, systemic blood flow; RA, right atrium; RV, right ventricle.

**Table 3:** Indices associated with impaired functional status (NYHA II-IV)

	OR (95% CI)	*P*
RA reservoir strain (per 1%)	0.91 (0.87-0.95)	0.006
Estimated RA pressure (per 1 mm Hg)	1.06 (1.01-1.11)	0.02
Doppler cardiac output (per 1 L/min/m^2^)	0.94 (0.83-1.01)	0.08

CI, confidence interval; NYHA, New York Heart Association; OR, odds ratio; RA, right atrium.

**Table 4:** Indices associated with hepatorenal function (MELD-XI score)

	β ± SE	*P*
RA reservoir strain (per 1%)	−0.31 ± 0.18	<0.001
Estimated RA pressure (per 1 mm Hg)	0.22 ± 0.13	0.007
Age (per 5 y)	0.08 ± 0.03	0.01

MELD-XI, model for end-stage liver disease excluding international normalized ratio; RA, right atrium; SE, standard error.

The patients were followed for 7 ± 3 years, and during this period, 11 (26%) patients had incident atrial flutter/tachycardia and 13 (30%) had incident atrial fibrillation, and the diagnoses of atrial arrhythmias were based on the electrocardiogram and Holter monitor in 18 and 6 cases, respectively. These 24 episodes of atrial arrhythmias occurred in 19 patients. Of the 24 episodes of atrial arrhythmias, 7 occurred in the setting of decompensated right heart failure, and all 7 patients underwent direct current cardioversion during hospitalization for heart failure. Of the other 17 episodes of atrial arrhythmia, 11 required direct current cardioversion, whereas the other 6 episodes terminated spontaneously. Overall, 12 of the 19 patients with atrial arrhythmias required rhythm control medical therapy alone (amiodarone n = 6, sotalol n = 4, dofetilide n = 2), whereas the other 7 patients required catheter ablation. The composite endpoint of atrial arrhythmia/heart failure hospitalization occurred in 19 (44%) patients. RA reservoir strain was independently associated with atrial arrhythmia/heart failure hospitalization (hazard ratio [HR]: 0.93 [0.89-0.97], *P* < 0.001) (Table 5). Of the 43 patients, 6 (14%) died from cardiovascular causes (end-stage heart failure n = 5 and sudden cardiac death n = 1) and 1 (2%) underwent heart transplant for end-stage heart failure. There was a trend toward an association between RA reservoir strain and the composite outcome of death/transplant (HR: 0.96 [0.92-1.00], *P* = 0.05) (Table 6).

**Table 5:** Indices associated with incident atrial arrhythmias and heart failure hospitalization

	HR (95% CI)	*P*
RA reservoir strain (per 1%)	0.93 (0.89-0.97)	<0.001
RA volume (per 1 mL/m^2^)	1.13 (1.06-1.21)	<0.001
Estimated RVSP (per 5 mm Hg)	1.06 (0.98-1.13)	0.07
Doppler cardiac output (per 1 L/min/m^2^)	0.96 (0.90-1.02)	0.1

CI, confidence interval; HR, hazard ratio; RA, right atrium; RVSP, right ventricular systolic pressure.

**Table 6:** Indices associated with cardiovascular death/transplant

	HR (95% CI)	*P*
RA reservoir strain (per 1%)	0.96 (0.92-1.00)	0.05
Estimated RA pressure (per 1 mm Hg)	1.04 (0.99-1.09)	0.07
Age (per 5 y)	1.03 (0.98-1.09)	0.09

CI, confidence interval; HR, hazard ratio; RA, right atrium.

### Exploratory analysis

Of the 43 patients, 34 (79%) had RA function assessment for 31 (27-44) months. There was a temporal decrease in RA reservoir strain from 25 ± 6% at baseline to 22 ± 7% during follow-up (Δ −3%, 95% confidence interval: −4% to −2%). The temporal decline in RA reservoir strain was independently associated with the composite endpoint of atrial arrhythmia/heart failure hospitalization (HR: 1.24 [1.16-1.32], *P* = 0.008 per unit decline in RA reservoir strain over time), independent of the RA reservoir strain on the baseline echocardiogram (Table 7).

**Table 7:** Indices associated with incident atrial arrhythmias and heart failure hospitalization in patients with serial echocardiograms (n = 34)

	HR (95% CI)	*P*
RA reservoir strain at baseline (per 1%)	0.95 (0.92-0.98)	0.004
ΔRA reservoir strain (per 1% decrease)	1.24 (1.16-1.32)	0.008
RA volume (per 1 mL/m^2^)	1.07 (1.01-1.13)	0.03
Estimated RVSP (per 5 mm Hg)	1.02 (0.91-1.14)	0.6
Doppler cardiac output (per 1 L/min/m^2^)	0.97 (0.88-1.09)	0.5

CI, confidence interval; HR, hazard ratio; RA, right atrium; RVSP, right ventricular systolic pressure.

## Discussion

In this study, we assessed RA function in adults with PA-IVS and biventricular repair and evaluated the relationship between RA function and clinical outcomes. The main findings are as follows: (1) assessment of RA function was feasible in 95% of the patients, and of these patients, RA dysfunction was nearly universal; (2) adults with PA-IVS had a high prevalence of functional impairment (67%) and hepatorenal dysfunction (49%), and a high incidence of atrial arrhythmia/heart failure (44%) and cardiovascular death (14%) during follow-up; (3) RA function was independently associated with clinical outcomes across multiple domains, and hence could be used for prognostication in this population.

Several studies have assessed clinical outcomes in adults with PA-IVS, and collectively these studies show that adults with PA-IVS had significant functional impairment, atrial arrhythmias, and mortality regardless of whether they underwent biventricular, univentricular, or 1.5 ventricular repair.^3^, ^4^, ^5^ These suboptimal outcomes have been linked to RV diastolic dysfunction, which in turn leads to right heart failure.^8^^,^^9^ Liang et al.^8^ demonstrated that patients with PA-IVS have reduced RV systolic and diastolic strain rate, and high RV late gadolinium enhancement score on cardiac magnetic resonance imaging, and that these indices of RV functional and structural remodelling were associated with peak oxygen consumption. A more recent study by To et al.^17^ showed that adults with PA-IVS had reduced RA reservoir, conduit, and booster strain, and that RA strain correlated with RV diastolic function indices. The current study builds on these previous data by demonstrating a link between RA dysfunction, as measured by RA reservoir strain, and clinical outcomes. To the best of our knowledge, this is the first study assessing the prognostic role of RA strain indices in this population.

There are robust haemodynamic and outcome data supporting the use of RA strain imaging for prognostication in patients with pulmonary arterial hypertension.^12^^,^^13^ Querejeta Roca et al.^12^ showed that RA reservoir strain reduced in patients with pulmonary arterial hypertension independent of RA size and RA pressure. Furthermore, RA dysfunction, as measured by RA strain, was an independent risk factor for heart failure hospitalization and all-cause mortality in patients with pulmonary arterial hypertension.^13^ These findings are consistent with the results of the current study.

Atrial function is complex and consists of a reservoir phase that occurs during atrial filling, a conduit phase that occurs during passive emptying of the atrium, and a pump phase that occurs during atrial systole.^12^, ^13^, ^14^ The reservoir function is dependent on atrial relaxation and annular descent, whereas the conduit and pump phases are dependent on ventricular relaxation and compliance. Patients with PA-IVS have intrinsic RV myocardial abnormalities that include myocyte disarray, abnormal capillary distribution, and fibrosis.^18^^,^^19^ The combination of these intrinsic histologic abnormalities and extrinsic factors such as pressure overload and cyanosis in infancy results in RV systolic and diastolic dysfunction, which in turn leads to RA dysfunction. Further, the additional volume load from tricuspid and pulmonary regurgitation, in the setting of a noncompliant RV, will lead to an increase in filling pressure and RA remodelling. We postulated that the complex interplay of these haemodynamic factors is responsible for the high prevalence of RA dysfunction observed in this cohort.

Another important finding from the current study was that the impact of RA remodelling and dysfunction was not just limited to the cardiovascular system but also affected the hepatorenal system. Hepatorenal dysfunction is a complication that has not been described in this population, and hence the novelty of this finding. It is noteworthy that half of the patients already had a MELD-XI score >11, which is a cutoff point that is associated with mortality in patients with congenital heart disease.^16^^,^^20^ Again, we postulate that hepatorenal dysfunction is driven by the high central venous pressure required to achieve adequate RV filling in the setting of RA and RV dysfunction.

The consistent association between RA dysfunction and adverse cardiovascular outcomes across multiple domains suggests that RA reservoir strain can be used to determine when to intensify therapy or consider transplant. Potential clinical applications include deciding on the timing of valve intervention or transplant evaluation in patients with deteriorating RA function and pressure, because the current data show that such patients have a significant risk of an adverse outcome. The clinical implication of hepatorenal dysfunction in this population is unclear and hence deserves further studies.

### Limitations

This is a single-centre retrospective study, and hence it is prone to referral and ascertainment bias. The small sample size did not allow for subgroup analyses. We did not have invasive haemodynamic indices in all the patients. In addition, we did not have cardiac magnetic resonance data and hence can only speculate about the role of diastolic dysfunction and atrial remodelling and fibrosis/scar. The data about coronary artery anomalies were not verified angiographically in all patients.

## Conclusions

Adults with PA-IVS and biventricular repair had a high prevalence of functional impairment and hepatorenal dysfunction, and high incidence of atrial arrhythmia/heart failure and cardiovascular death. The assessment of RA function by strain imaging was feasible in almost all patients and was associated with clinical outcomes. Collectively, these data suggest that RA strain indices can be used for risk stratification in this population. Further studies are needed to determine the prognostic implications of hepatorenal dysfunction, and whether using RA function indices for clinical decision making will lead to improved outcomes in this population.

## Acknowledgement

We thank James Welper for performing offline image analysis for this study.

## Ethics Statement

The study was approved by the Mayo Clinic Institutional Review Board, and adhere to ethical recommendations from the institution.

## Contributor’s Statement

ACE was responsible for the planning, conduct, reporting, drafting, and critical review of the manuscript and takes responsibility for the overall content. WRM and HMC were responsible for the critical review of the manuscript.

## Funding Sources

ACE is supported by 10.13039/100000050National Heart, Lung, and Blood Institute (NHLBI) grant K23 HL141448. The MACHD Registry is supported by the Al-Bahar Research grant.

## Disclosures

The authors have no conflicts of interest to disclose.
